# Supplementary material for: G3BP1 controls the senescence-associated secretome and its impact on cancer progression
Source: Nat Commun. 2020 Oct 5;11:4979. doi: 10.1038/s41467-020-18734-9 (PMC7536198; doi:10.1038/s41467-020-18734-9)
Supplement: Supplementary file 1 — Supplementary Information [file 41467_2020_18734_MOESM1_ESM.pdf]

The stress granule protein G3BP1 controls the senescence associated secretome and its impact on cancer progression

Omer et al.

Supplementary Information

This file contains Supplementary Figures 1-14 with figure legends.

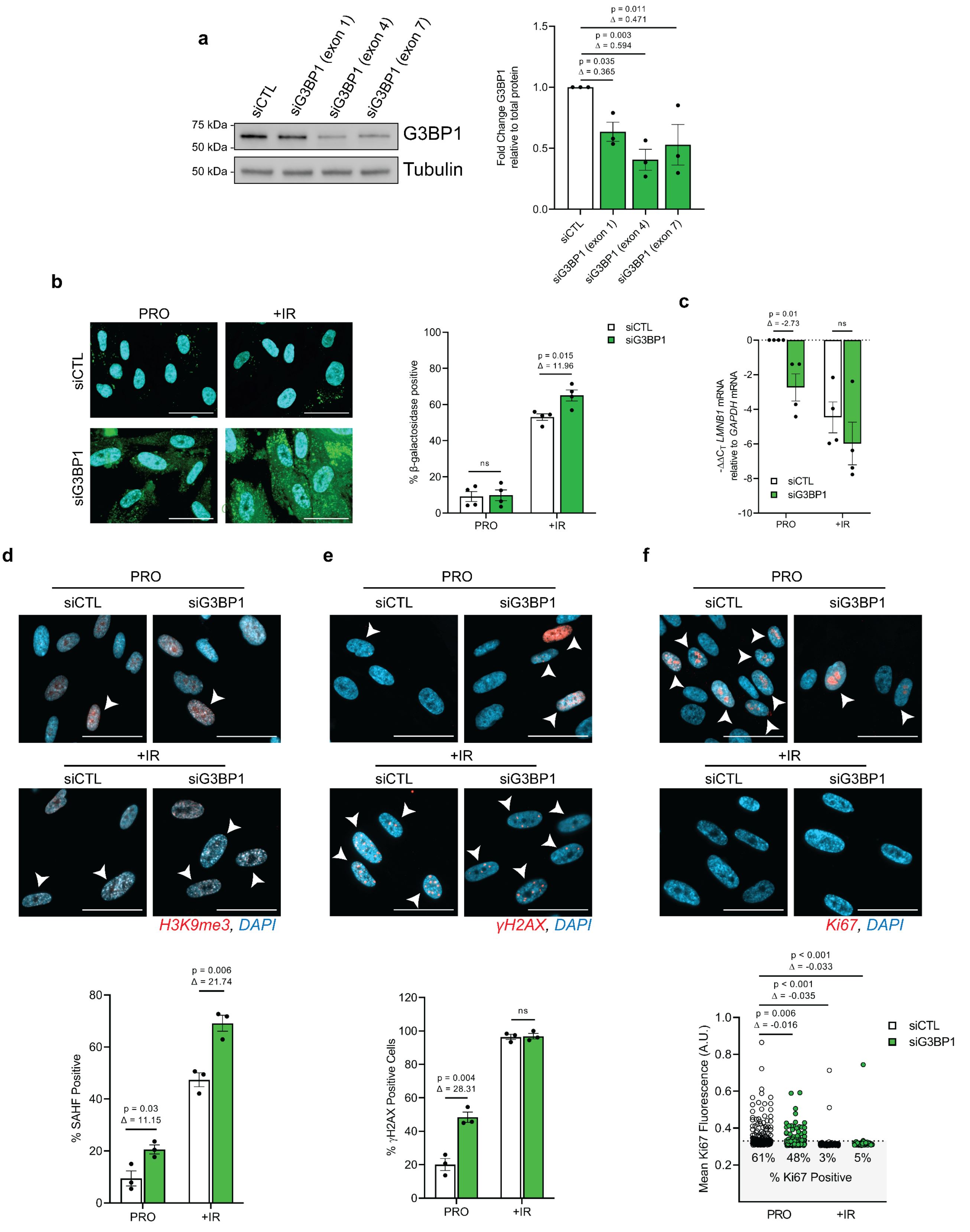

Supplementary Figure 1: G3BP1 depletion increases abundance of senescent cells and senescence associated phenotypes in IMR-90 cells. a, (left) Cell lysates from proliferative WI-38 cells treated with siRNA targeting three different exons of G3BP1 and

scrambled control (siCTL) were subjected to western blot analysis against indicated proteins. (right) Quantifications represent a mean of relative protein levels from three independent experiments ± s.e.m (two-tailed unpaired student's t-test). **b**, (left) Proliferative (PRO) and post ionizing radiation (+IR) IMR-90 cells were treated with siRNA against G3BP1 (siG3BP1) or scrambled control (siCTL). Cells were treated with C12FDG to assess SA-β-gal activity and treated with DAPI to visualize nuclei during PRO and +IR. Scale bar, 50 μm. (right) Graph representing % SA-β-gal positive cells in (left). The data is a mean of three independent experiments ± s.e.m. (two-tailed unpaired student's t-test). **c,** RNA was extracted from IMR-90 cells during PRO and +IR and assayed by RT-qPCR using primers against *LMNB1* mRNA**.** The data is a mean of four independent experiments ± s.e.m (unpaired student's t-test). **d-f,** (top) WI-38 cells were analyzed by immunofluorescence against (**d**) H3K9me3, (**e**) γH2AX and (**f**) Ki67 during PRO and +IR. DAPI staining was used to visualize nuclei. Arrows indicate cells positive for indicated marker. Scale bar, 50 μm. (bottom) Graphs representing (**d**) % SAHF positive cells determined by formation of DAPI foci colocalized with H3K9me3 foci, (**e**) % γH2AX positive cells, determined by the presence of >4 nuclear γH2AX foci >300nm in diameter, and (**f**) mean Ki67 intensity. The data is a mean three independent experiments ± s.e.m, for % SAHF positive cells and % γH2AX positive cells, and a distribution of a minimum of 150 cells per condition, n = 230, 174, 172, 155 from left to right (two-tailed unpaired student's t-test, exact <0.001 p-values from left to right: 3.2E-9, 6.8E-8). Source Data for all the graphs are provided in the Source Data File.

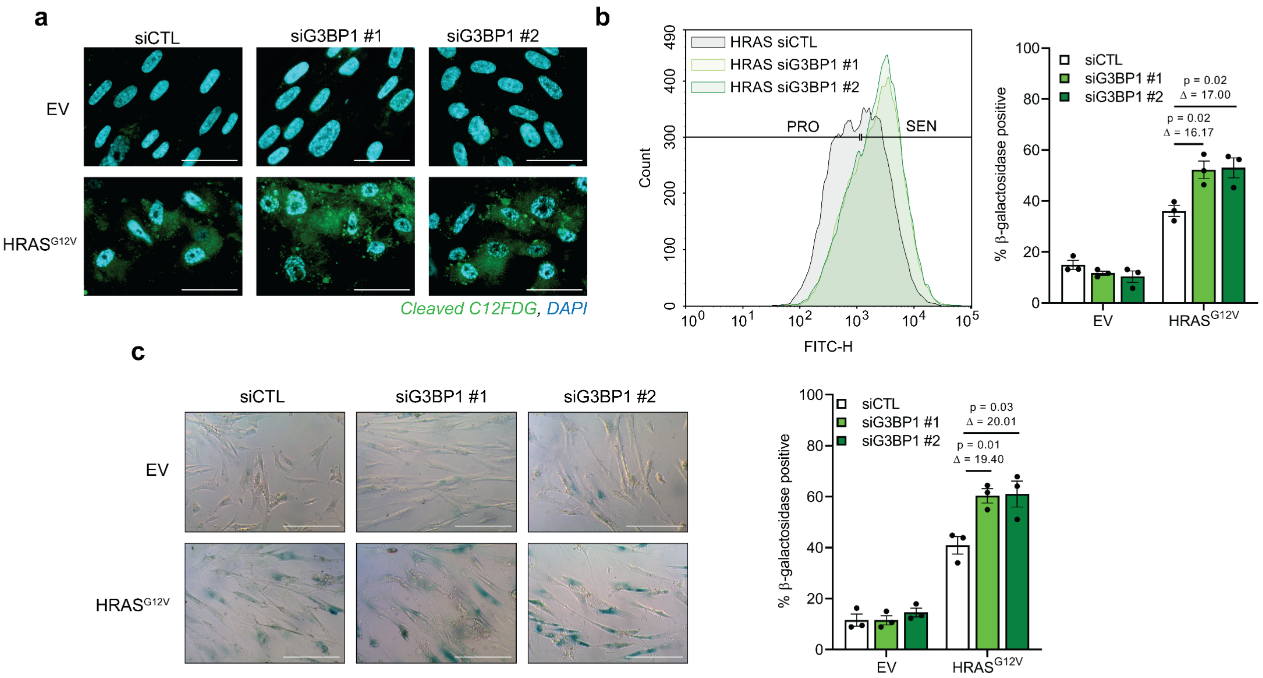

Supplementary Figure 2: G3BP1 depletion increases abundance of senescent cells during oncogene induced senescence in WI-38 cells. WI-38 cells were treated with siRNA against G3BP1 (siG3BP1 #1 and #2) or scrambled control (siCTL) and then transduced with empty vector (EV) or HRAS ($HRAS^{G12V}$). Senescence was then assessed seven-days post-transduction with either EV or $HRAS^{G12V}$**.** **a**, SA-β-gal was assessed using C12FDG. Cells were treated with DAPI to visualize nuclei. Scale bar, 50 μm. **b**, (left) SA-β-gal was assessed using C12FDG via flow cytometer. (right) Graph representing % SA-β-gal positive cells using flow cytometer. The data is a mean of three independent experiments ± s.e.m. (two-tailed unpaired student's t-test). **c**, (left) SA-β-gal was assessed using X-gal. Scale bar, 100 μm. (right) Graph representing % SA-β-gal positive cells measured by flow cytometry. The data is a mean of three independent experiments ± s.e.m. (two-tailed unpaired student's t-test). Source Data for all the graphs are provided in the Source Data File.

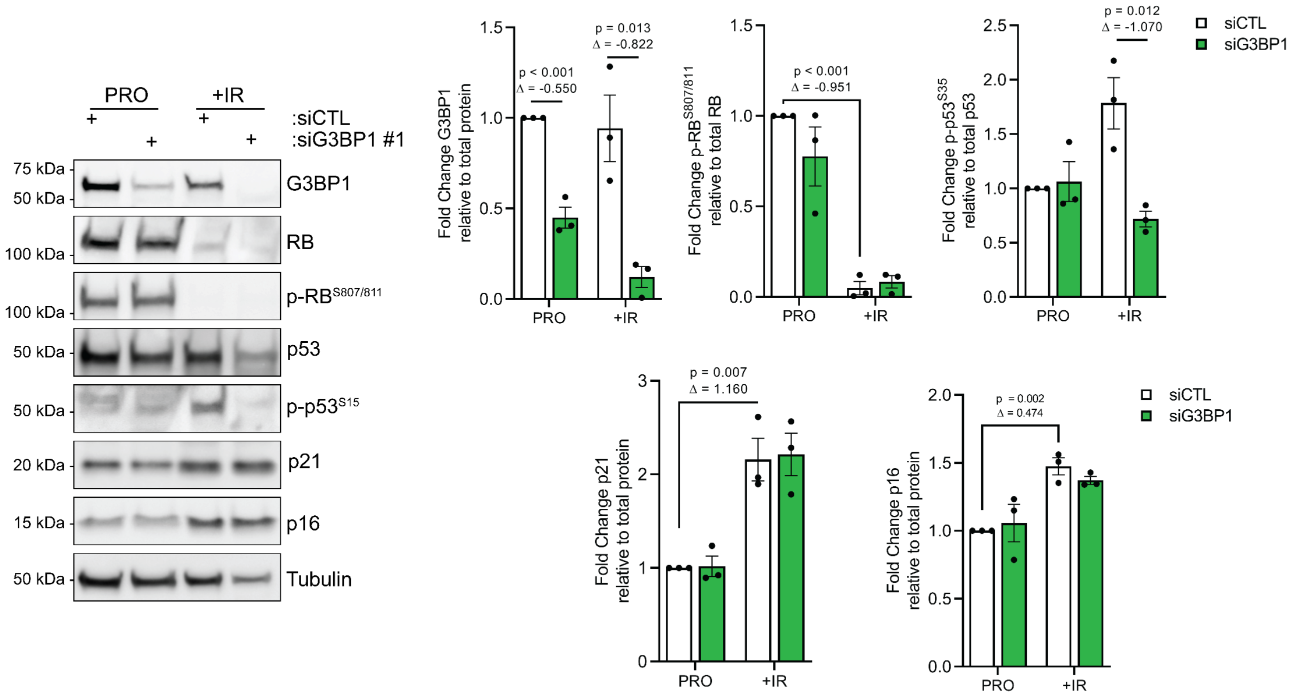

Supplementary Figure 3: G3BP1 depletion increases senescence associated phenotypes but not levels of cell cycle regulators in irradiated IMR-90 cells. Cell lysates from proliferative (PRO) and eight-days post ionizing radiation (+IR) IMR-90 cells were subjected to western blot analysis against indicated proteins. Quantifications represent a mean of relative protein levels from three independent experiments ± s.e.m (two-tailed unpaired student's t-test, exact <0.001 p-values from left to right: 0.00066, 0.00001). Source Data for all the graphs are provided in the Source Data File.

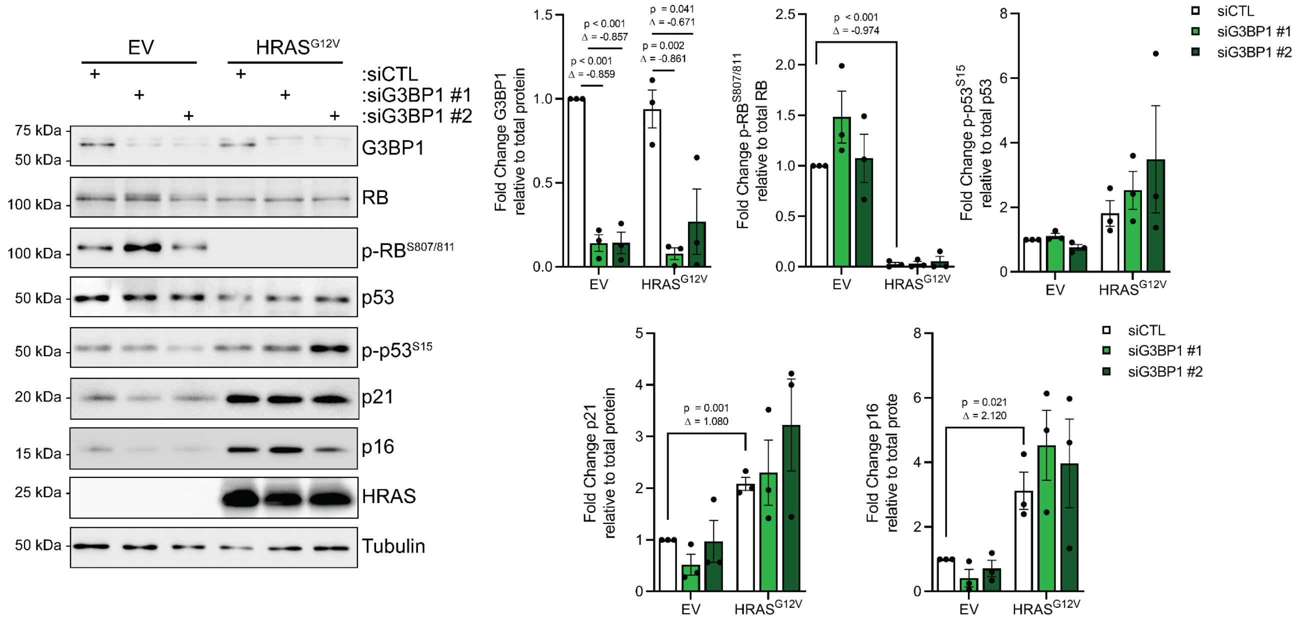

Supplementary Figure 4: G3BP1 does not affect levels of established cell cycle regulators during oncogene-induced senescence in WI-38 cells. WI-38 cells were treated with siRNA against G3BP1 (siG3BP1 #1 and #2) or scrambled control (siCTL) and assessed during proliferative cells transduced with empty vector (EV) and seven-days post-transduction with HRAS (HRAS^{G12V}). (left) Cell lysates were subjected to western blot analysis against indicated proteins. (right) Quantifications represent a mean of relative protein levels from three independent experiments ± s.e.m (two-tailed unpaired student's t-test, exact <0.001 p-values from left to right: 0.00001, 0.00084, 5.0E-7). Source Data for all the graphs are provided in the Source Data File.

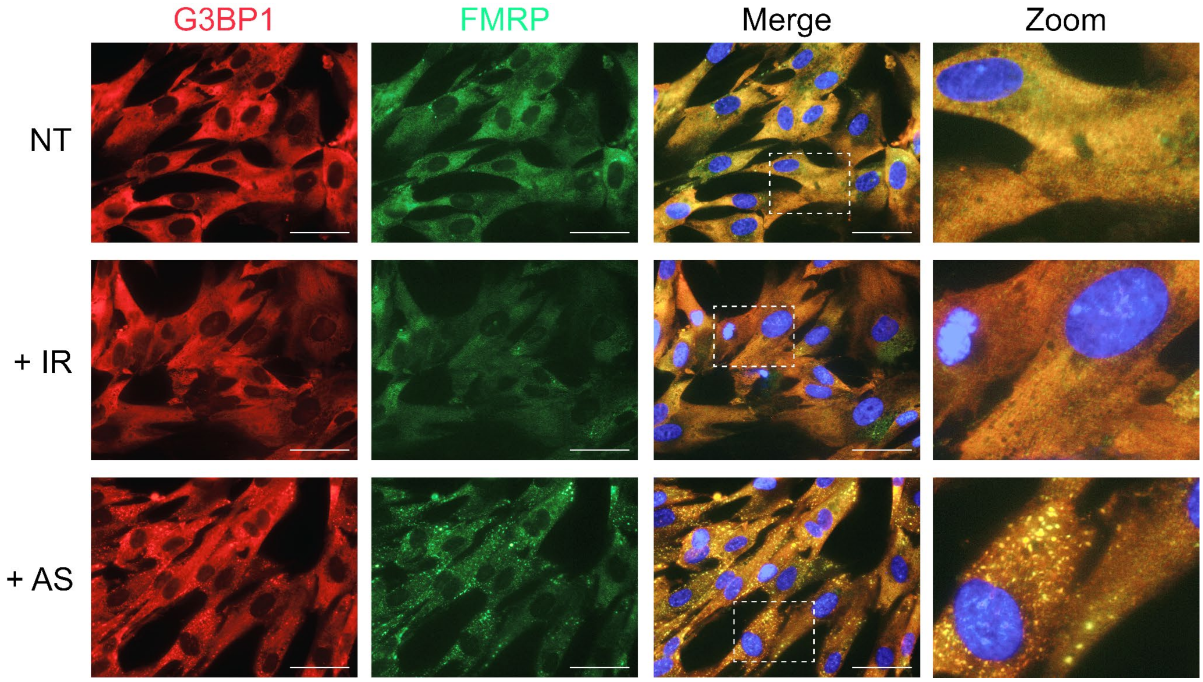

Supplementary Figure 5: Irradiation does not induce stress granule formation. WI-38 cells were treated or not with ionizing radiation (+IR). Cells were fixed 15 minutes after irradiation and used for immunofluorescence to assess the presence of well-known stress granules markers such as G3BP1 and FMRP. Scale bar, 50 μm.

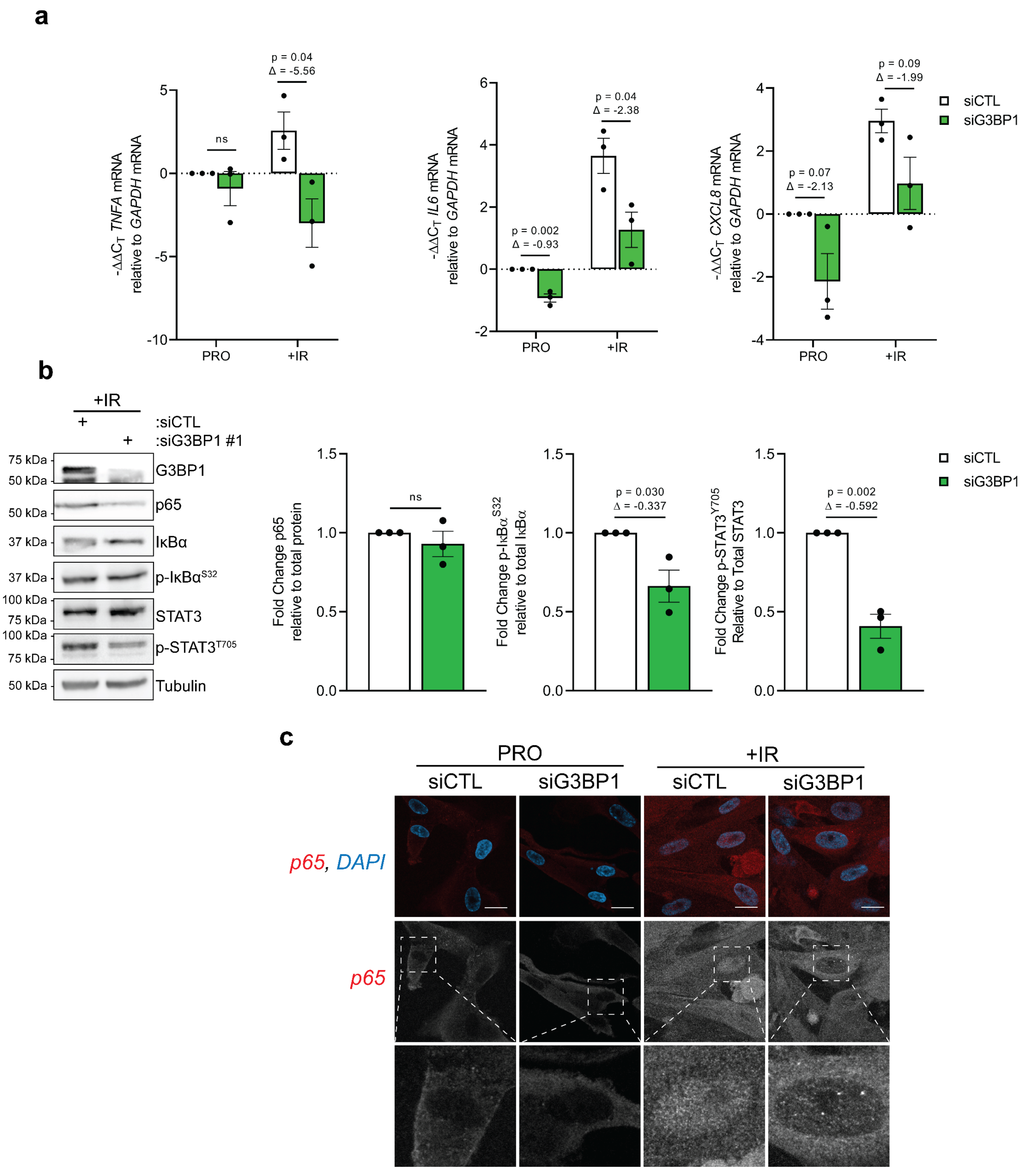

Supplementary Figure 6: Depletion of G3BP1 impairs canonical SASP signaling through reduction of STAT3 and NF-κB signaling in IMR-90 cells. a, RNA was extracted from cells during proliferative stage (PRO) and eight-days post ionizing radiation (+IR) from IMR-90 cells treated with siRNA against G3BP1 (siG3BP1) or scrambled control (siCTL) and assayed by RT-qPCR using primers against indicated mRNA**.** The data is a mean of three independent experiments ± s.e.m (two-tailed unpaired student's t-test). **b,** Cell lysates from PRO and +IR IMR-90 cells were subjected to western blot analysis against indicated proteins.

(right) Quantifications represent a mean of relative protein levels from three independent experiments ± s.e.m (two-tailed unpaired student's t-test). **c,** IMR-90 cells were analyzed by immunofluorescence against p65 in PRO and +IR IMR-90 cells. DAPI staining was used to visualize nuclei. Scale bar, 20 μm. Source Data for all the graphs are provided in the Source Data File.

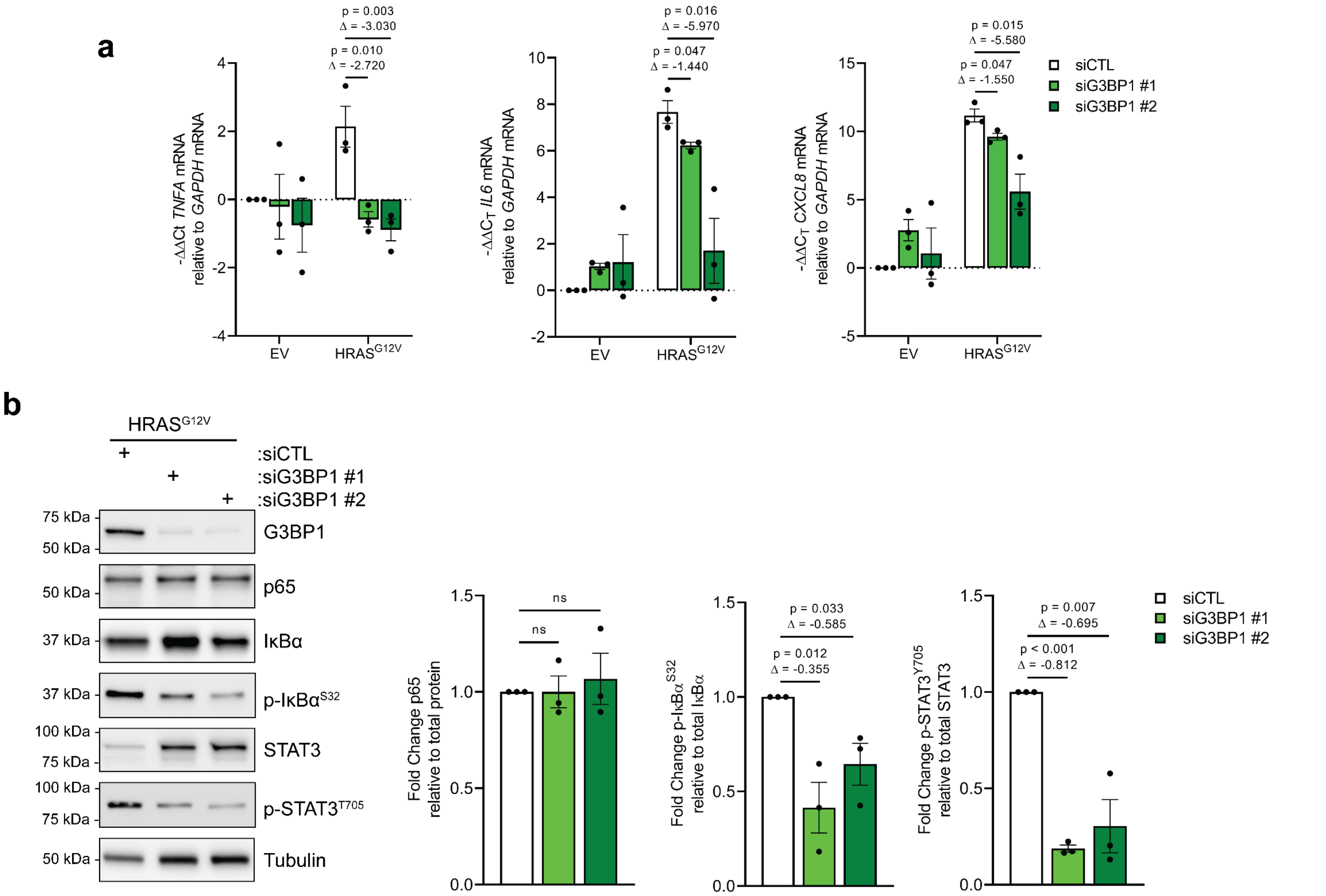

Supplementary Figure 7: Depletion of G3BP1 impairs canonical inflammatory SASP expression through reduced activation of STAT3 and NF-κB signaling during oncogene-induced senescence in WI-38 cells. WI-38 cells were treated with siRNA against G3BP1 (siG3BP1 #1 and #2) or scrambled control (siCTL) and assessed in cells transduced with empty vector (EV) and seven-days post-transduction with HRAS ($HRAS^{G12V}$). **a**, RNA was extracted and assayed by RT-qPCR using primers against indicated mRNA**.** The data is a mean of three independent experiments ± s.e.m (two-tailed unpaired student's t-test). **b,** (left) Cell lysates were subjected to western blot analysis against indicated proteins. (right) Quantifications represent a mean of relative protein levels from three independent experiments ± s.e.m (two-tailed unpaired student's t-test, exact <0.001 p-values from left to right: 0.0007). Source Data for all the graphs are provided in the Source Data File.

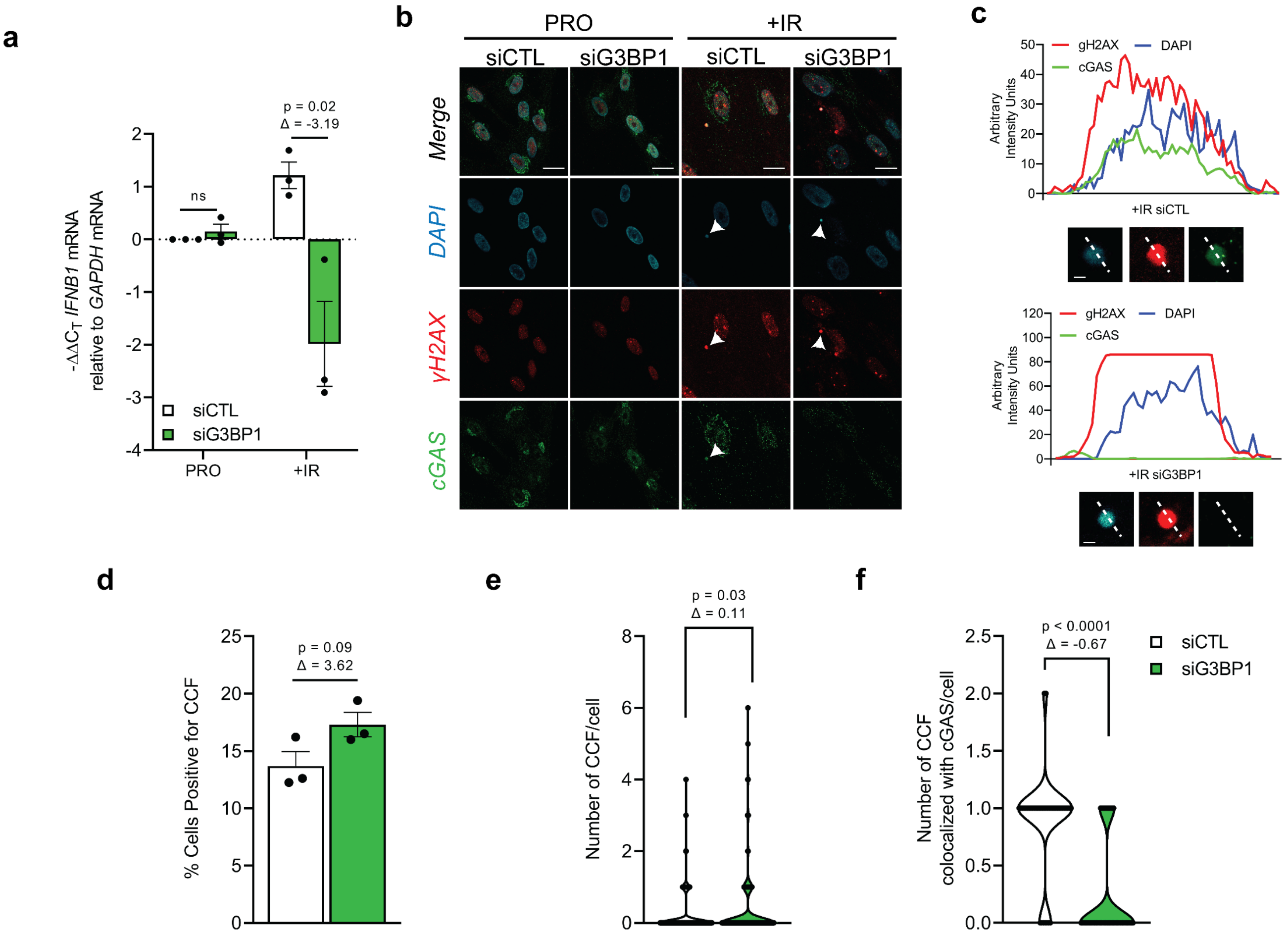

Supplementary Figure 8: G3BP1 loss inhibits cGAS signaling in senescent cells required for activation of SASP in irradiated IMR-90 cells. a, RNA was extracted from cells during proliferative stage (PRO) and eight-days post ionizing radiation (+IR) from IMR-90 cells treated with siRNA against G3BP1 (siG3BP1) or scrambled control (siCTL) and assayed by qPCR using primers against *IFNB1* mRNA. The data is a mean of three independent experiments ± s.e.m (two-tailed unpaired student's t-test). **b,** IMR-90 cells were analyzed by immunofluorescence against γH2AX and cGAS during PRO and SEN. DAPI staining was used to visualize nuclei. White arrows indicate CCFs. Scale bar, 20 μm. **C,** Graphs represent intensity profile for CCFs in SEN siCTL (**top**) and SEN siG3BP1 (**bottom**) for γH2AX, cGAS, and DAPI. Plot is representative of foci shown below. Scale bar, 2 μm. **d, e, f,** Graph of % SEN cells positive for CCF ± s.e.m (**d**), number of CCFs present in SEN cells (**e**), and number of CCFs present in SEN cells colocalized with cGAS (**f**). The data is representative of three independent experiments (two-tailed unpaired student's t-test, exact <0.001 p-values from left to right: 7.6E-11). Source Data for all the graphs are provided in the Source Data File.

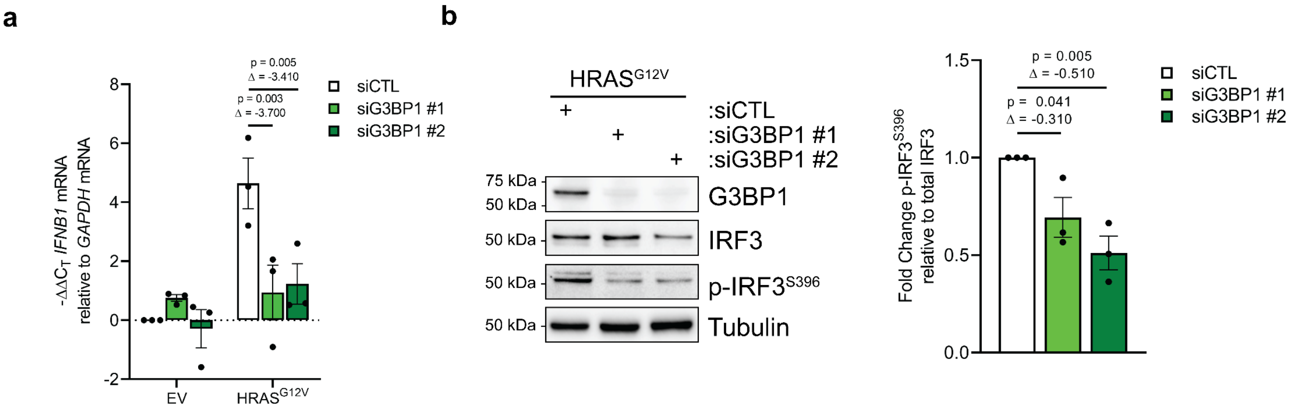

Supplementary Figure 9: G3BP1 depletion inhibits cGAS signaling in senescent cells required for activation of SASP during oncogene-induced senescence in WI-38 cells. WI-38 cells were treated with siRNA against G3BP1 (siG3BP1 #1 and #2) or scrambled control (siCTL) and assessed in cells transduced with empty vector (EV) or seven-days post-transduction with HRAS ($HRAS^{G12V}$). **a**, RNA was extracted and assayed by qPCR using primers against *IFNB1* mRNA**.** The data is a mean of three independent experiments ± s.e.m (two-tailed unpaired student's t-test). **b,** (left) Cell lysates were subjected to western blot analysis against indicated proteins. (right) Quantifications represent a mean of relative protein levels from three independent experiments ± s.e.m (two-tailed unpaired student's t-test). Source Data for all the graphs are provided in the Source Data File.

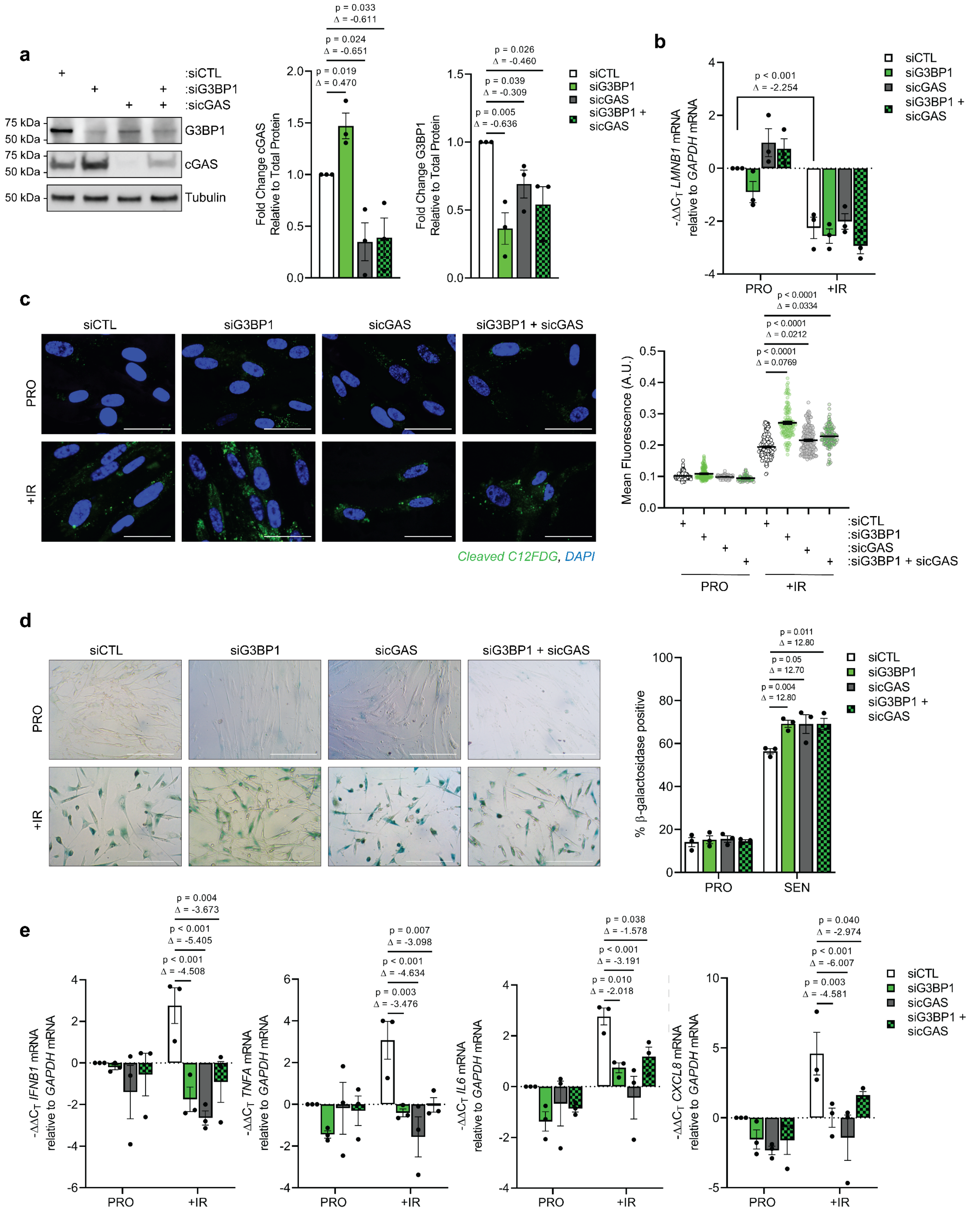

Supplementary Figure 10: Knockdown of cGAS recapitulates the effects of G3BP1 depletion on SASP induction without preventing cellular senescence. WI-38 cells were

treated with siRNA against G3BP1 (siG3BP1) and/or cGAS (sicGAS) or scrambled control (siCTL) and assessed during proliferative stage (PRO) and eight-days post-ionizing radiation (+IR). **a,** (left) Cell lysates obtained from PRO cells were analysed by western blot against indicated proteins. (right) Quantifications represent a mean of relative protein levels from three independent experiments ± s.e.m (two-tailed unpaired student's t-test). **b,** RNA was extracted from WI-38 cells during PRO stage and eight-days post ionizing radiation (+IR) and assayed by RT- qPCR using primers against *LMNB1* mRNA**.** The data is a mean of three independent experiments ± s.e.m (two-tailed unpaired student's t-test). **c,** (left) SA-β-gal was assessed using C12FDG. Cells were treated with DAPI to visualize nuclei. Scale bar, 50 µm. (right) Mean fluorescence from (left) was measured. The data is measured from a minimum of 50 cells, the mean is shown ± s.e.m, n = 289, 159, 133, 59, 184, 125, 186, 117 (one-way ANOVA, Fisher's LSD). **d,** (left) SA-β-gal was assessed using X-gal. Scale bar, 100 µm. (right) Graph representing % SA-β-gal positive cells from (**left**). The data is a mean of three independent experiments ± s.e.m. (two-tailed unpaired student's t-test, exact <0.001 p-values from left to right: 1.0E-15, 2.5E-12, 1.0E-15). **e,** RNA was extracted and assayed by RT-qPCR using primers against the indicated mRNA**.** The data is a mean of three independent experiments ± s.e.m (two-way ANOVA, Fisher's LSD, exact <0.001 p-values from left to right: 0.0009, 0.0002, 0.0003, 0.0003, 0.0004). Source Data for all the graphs are provided in the Source Data File.

a

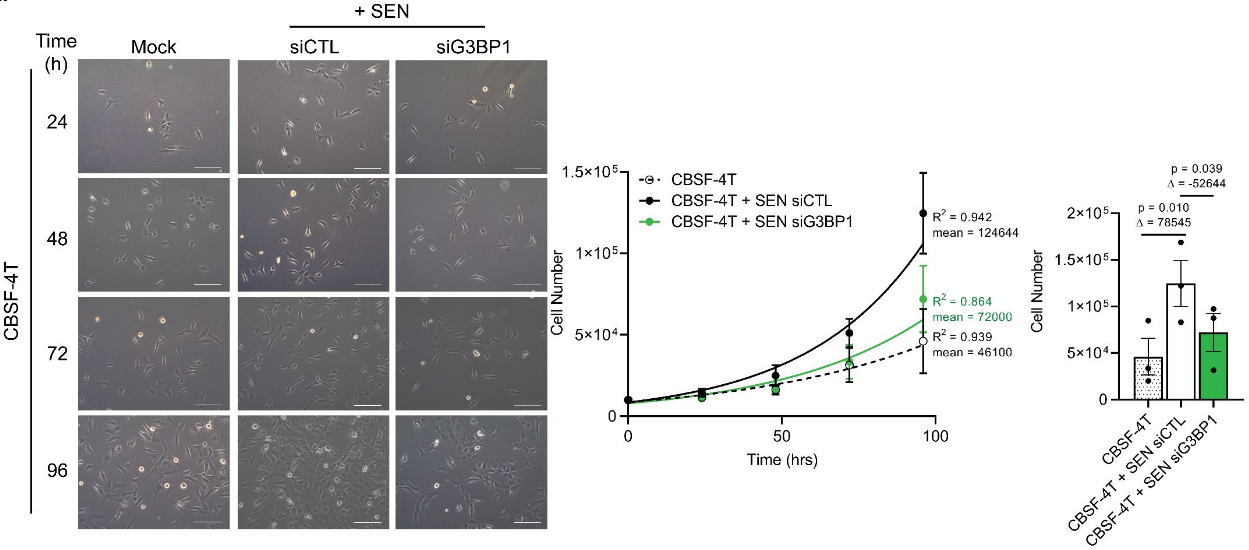

Supplementary Figure 11: SASPless G3BP1 depleted senescent cells do not promote senescence-associated cell growth of immortalized skin fibroblasts *in vitro.* (left) Images of CBS-4T skin fibroblasts cells without co-culture or with co-culture of WI-38 cells eight-days post ionizing radiation (SEN) cells treated with siRNA against G3BP1 (siG3BP1) or scrambled control (siCTL). Scale bar, 200 µm. (middle) Graph representing total number of CBSF-4T cells quantified over time. The data is a mean of three independent experiments ± s.e.m (nonlinear regression, R-squared values). (right) Graph representing total number of CBSF-4T cells quantified after 96 hours as in (middle). The data is a mean of three independent experiments ± s.e.m (one-way ANOVA, Fisher’s LSD). Source Data for all the graphs are provided in the Source Data File.

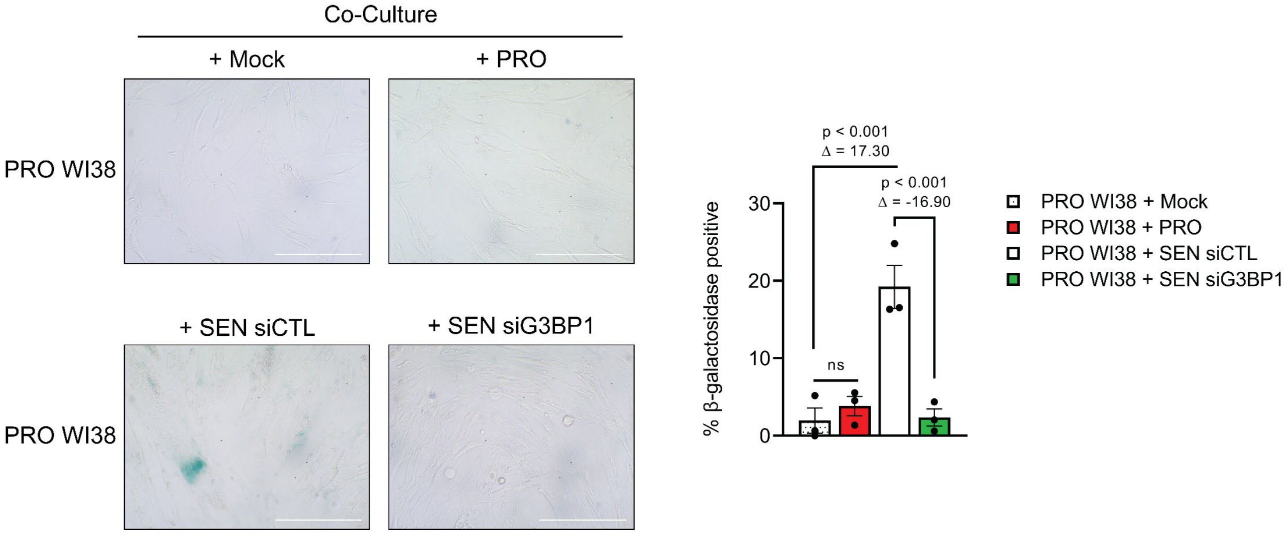

Supplementary Figure 12: SASPless G3BP1 depleted senescent cells do not promote paracrine senescence *in vitro*. WI-38 proliferative cells were co-cultured with Mock, or proliferative WI-38 cells, or cells treated with siRNA against G3BP1 (siG3BP1) and scrambled control (siCTL) eight-days post-ionizing radiation (+IR). (left) SA-β-gal was assessed using X-gal. Scale bar, 100 µm. (right) Graph representing % SA-β-gal positive cells from (left). The data is a mean of three independent experiments ± s.e.m. (one-way ANOVA, Fisher's LSD, exact <0.001 p-values from left to right: 0.0001, 0.0002). Source Data for the graph are provided in the Source Data File.

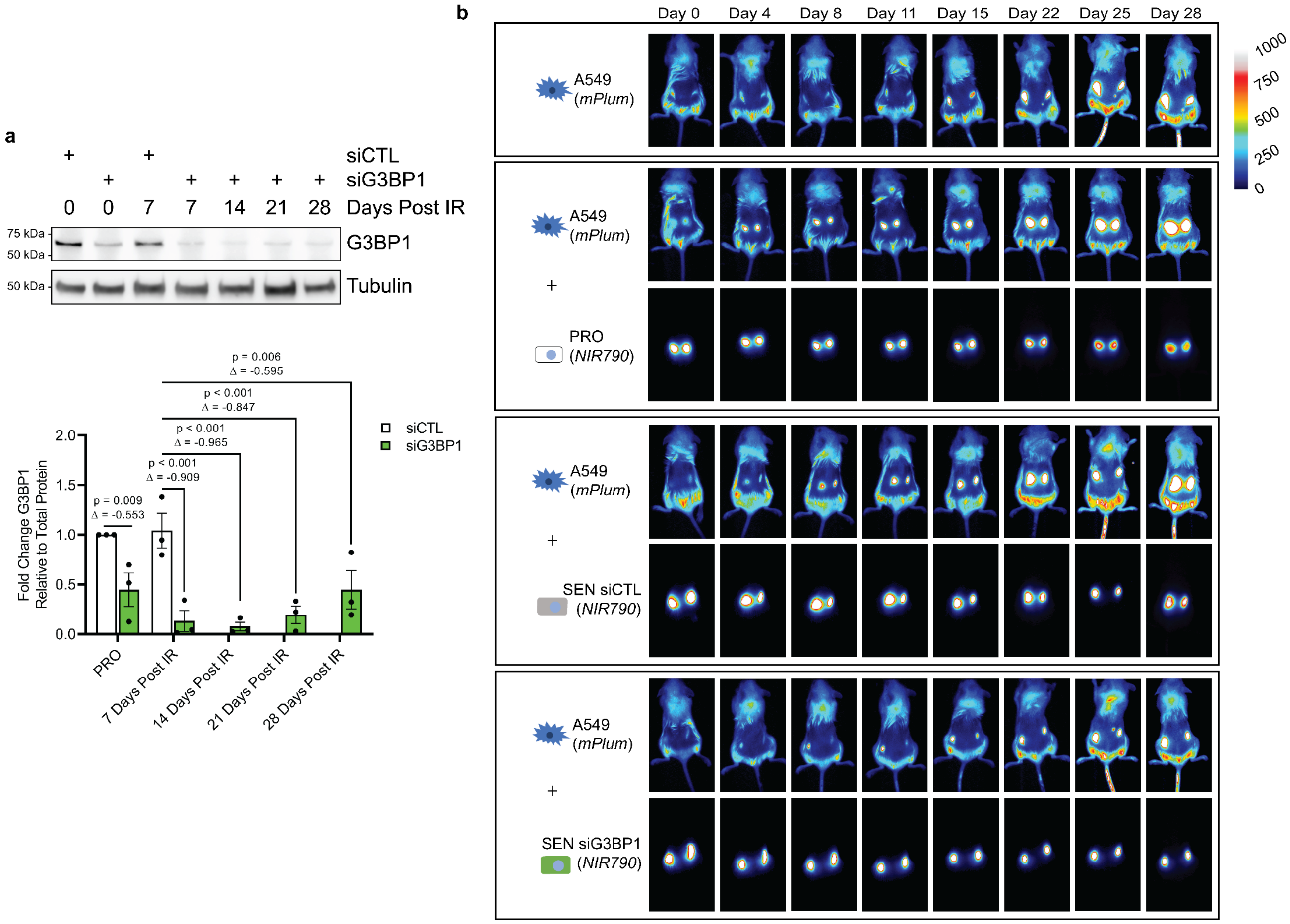

Supplementary Figure 13: Time course imagery of co-injected senescent cells depleted of G3BP1 with A549 tumorigenic cells. a, (top) Cell lysates from proliferative or irradiated cells treated with siRNA targeting G3BP1 (siG3BP1) or scrambled control (siCTL) were collected at the indicated time points and analyzed using western blot against indicated proteins. (bottom) Quantifications represent a mean of relative protein levels from three independent experiments ± s.e.m (one-way ANOVA, Fisher's LSD, exact <0.001 p-values from left to right: 0.0002, 0.0001, 0.0004). **b,** Images of fluorescent signal from A549 injected alone (expressing *mPlum*), co-injected A549 cells (expressing *mPlum*) and proliferative WI-38 cells treated with siRNA targeting scrambled control (stained with NIR790), co-injected A549 cells (expressing *mPlum*) and senescent WI-38 cells treated with siRNA targeting scrambled control (stained with NIR790), and co-injected A549 cells (expressing *mPlum*) and senescent WI-38 cells treated with siRNA targeting G3BP1 (stained with NIR790) were obtained using the Q-Lumi In Vivo imaging system. Images from day 0, day 4, day 8, day 11, day 15, day 22, day 25 and day 28 are shown. Source Data for the graph in a are provided in the Source Data File.

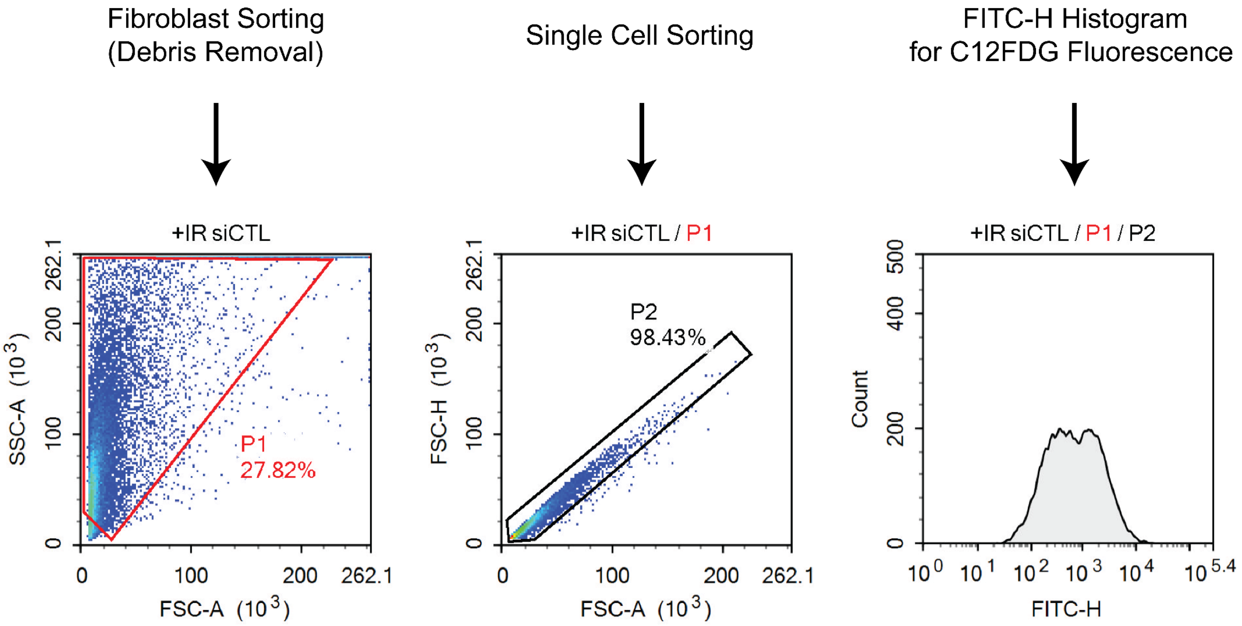

Sample Statistics of +IR siCTL

Gate	Count	% Parent	X	Y	Median X	Median Y
All	50,000					
└ P1	13,908	27.82%	FSC-A	SSC-A	13,247	71,857
└ P2	13,690	98.43%	FSC-A	FSC-H	13,324	11,203

Supplementary Figure 14: Gating strategy for Flow Cytometry analysis. Gating strategy for flow cytometry analysis of senescent WI-38 cells. The above gating strategy corresponds to flow cytometry data from Fig. 1b and Supplementary Fig. 2b. To estimate relative SA-β-Gal activity, based on cleaved C12FDG fluorescence, a two-parameter display of FSC vs. SSC was set up excluding subcellular debris. Single cells were selected using a two-parameter display of FSC-H vs. FSC-A. Non-labeled samples were used to determine auto-fluorescence.
