# Supplementary material for: G3BP1 controls the senescence-associated secretome and its impact on cancer progression
Source: Nat Commun. 2020 Oct 5;11:4979. doi: 10.1038/s41467-020-18734-9 (PMC7536198; doi:10.1038/s41467-020-18734-9)
Supplement: Supplementary file 3 — Description of Additional Supplementary Files [file 41467_2020_18734_MOESM3_ESM.pdf]

Description of Additional Supplementary Files

File Name: Supplementary Data 1
Description: **Differential Expression Analysis and Ingenuity Pathway Analysis of RNA Sequencing Data of senescent WI-38 cells expressing or not G3BP1.** Total RNA from post ionizing radiation (SEN) WI-38 cells treated with siRNA against G3BP1 (siG3BP1) or scrambled control (siCTL) were subjected to RNA sequencing. Differential expression analysis and ingenuity pathway analysis are shown.

File Name: Supplementary Data 2
Description: **Raw Multiplex Analysis of conditioned medium from senescent WI-38 cells expressing or not G3BP1.** Conditioned media from PRO and from +IR WI-38 cells treated with siRNA against G3BP1 (siG3BP1) and scrambled control (siCTL) were analyzed by multiplex arrays. The raw data from three independent experiments is shown.

File Name: Supplementary Data 3
Description: **Raw Multiplex Analysis of conditioned medium from senescent WI-38 cells treated with or without EGCG.** Conditioned media from proliferative (PRO) cells and eight-day post ionizing radiation (SEN) WI-38 cells treated with or without 40μM EGCG. The heatmap indicates the fold change in comparison to the control PRO, SEN, and SEN treated with 40μM EGCG. The raw data from three independent experiments is shown.
