# Supplementary material for: G3BP1 controls the senescence-associated secretome and its impact on cancer progression
Source: Nat Commun. 2020 Oct 5;11:4979. doi: 10.1038/s41467-020-18734-9 (PMC7536198; doi:10.1038/s41467-020-18734-9)
Supplement: Supplementary file 7 — Reporting Summary [file 41467_2020_18734_MOESM7_ESM.pdf]

nature research

Corresponding author(s): Imed-Eddine Gallouzi

Last updated by author(s): Aug 19, 2020

Reporting Summary

Nature Research wishes to improve the reproducibility of the work that we publish. This form provides structure for consistency and transparency in reporting. For further information on Nature Research policies, see Authors & Referees and the Editorial Policy Checklist.

Statistics

For all statistical analyses, confirm that the following items are present in the figure legend, table legend, main text, or Methods section.

n/a	Confirmed	
☐	☒	The exact sample size (*n*) for each experimental group/condition, given as a discrete number and unit of measurement
☐	☒	A statement on whether measurements were taken from distinct samples or whether the same sample was measured repeatedly
☐	☒	The statistical test(s) used AND whether they are one- or two-sided<br>*Only common tests should be described solely by name; describe more complex techniques in the Methods section.*
☒	☐	A description of all covariates tested
☐	☒	A description of any assumptions or corrections, such as tests of normality and adjustment for multiple comparisons
☐	☒	A full description of the statistical parameters including central tendency (e.g. means) or other basic estimates (e.g. regression coefficient) AND variation (e.g. standard deviation) or associated estimates of uncertainty (e.g. confidence intervals)
☐	☒	For null hypothesis testing, the test statistic (e.g. *F*, *t*, *r*) with confidence intervals, effect sizes, degrees of freedom and *P* value noted<br>*Give P values as exact values whenever suitable.*
☒	☐	For Bayesian analysis, information on the choice of priors and Markov chain Monte Carlo settings
☒	☐	For hierarchical and complex designs, identification of the appropriate level for tests and full reporting of outcomes
☒	☐	Estimates of effect sizes (e.g. Cohen's *d*, Pearson's *r*), indicating how they were calculated

Our web collection on statistics for biologists contains articles on many of the points above.

Software and code

Policy information about availability of computer code

Data collection	Flow Cytometry: FACSDiva 8 was used during collection<br>RNA-Seq: RNA-seq libraries were sequenced on the Illumina NextSeq 500 platform at the Institute for Research in Immunology and Cancer (IRIC) Genomics Core Facility, University of Montreal, to produce over 60 million, 100 nucleotide paired-end reads per sample. Sequences were trimmed for sequencing adapters and low quality 3' bases using Trimmomatic version 0.35 and aligned to the reference human genome version GRCh38 (gene annotation from Gencode version 26, based on Ensembl 88) using STAR version 2.5.1b. Gene expressions were obtained both as readcount directly from STAR as well as computed using RSEM in order to obtain gene and transcript level expression, either in TPM or FPKM values, for these stranded RNA libraries. DESeq2 version 1.22.1 was then used to normalize gene readcounts.
Data analysis	Ingenuity Pathway Analysis (IPA) (Version 01-14 (01-14)) Qiagen<br>GraphPad Prism 7.0 was used for statistical analyses<br>FlowJo Version10.5.2 was used to analyze Flow Cytometry Data<br>Image Lab 6.0.1 was used to analyze Western Blot Data<br>Cell Profiler 3.1.9 was used to analyze Fluorescent Microscopy Data

For manuscripts utilizing custom algorithms or software that are central to the research but not yet described in published literature, software must be made available to editors/reviewers. We strongly encourage code deposition in a community repository (e.g. GitHub). See the Nature Research guidelines for submitting code & software for further information.

Data

Policy information about availability of data

All manuscripts must include a data availability statement. This statement should provide the following information, where applicable:

- Accession codes, unique identifiers, or web links for publicly available datasets
- A list of figures that have associated raw data
- A description of any restrictions on data availability

The data reported in this study in support of all the findings outlined are available from the corresponding author upon reasonable request. The source data underlying Figs. 1, 3-7 and Supplementary Figs. 1–4 and 6–13 are provided as a Source Data file. The raw multiplex analysis data for Figs. 3b and 5c are available in the Supplementary Data 2 and 3 files, respectively. The raw RNASeq data is available in the Supplementary Data 1 file and have been deposited into NCBI Gene Expression Omnibus (GEO) database under accession number GSE151745. For RNA Sequencing Analysis, sequences were aligned to the reference human genome version GRCh38 (gene annotation from Gencode version 26, based on Ensembl 88) using STAR version 2.5.1b.

Field-specific reporting

Please select the one below that is the best fit for your research. If you are not sure, read the appropriate sections before making your selection.

☒ Life sciences ☐ Behavioural & social sciences ☐ Ecological, evolutionary & environmental sciences

For a reference copy of the document with all sections, see nature.com/documents/nr-reporting-summary-flat.pdf

Life sciences study design

All studies must disclose on these points even when the disclosure is negative.

Sample size	No sample-size calculations were performed. When working with experimental repeats of the same biological sample (Western, RT-qPCR, Flow Cytometry and Immunofluorescence), 3 samples were used per group for minimal statistics requirements.<br><br>For RNA sequencing, we used 2 samples per group which is the minimum required for statistical analysis by DESeq2.<br><br>For in vivo studies, sample size was determined to be adequate based on the magnitude and consistency of measurable differences between groups.
Data exclusions	No data were excluded
Replication	Replicate experiments were successful. All experiments were replicated independently a minimum of 3 times as noted in each figure.
Randomization	When working with experimental repeats of the same biological sample, each treatment was paired with the respective controls, and the same setting was repeated independently several times.<br><br>For in vivo experiments all groups were compromised of 9-week old NOD/SCID/IL2Rγ null (NSG) Female Mice. Similarly to above, mice were grouped based on treatment and were randomly assigned into groups.
Blinding	No blinding was done during experimentation since no clinically relevant experiments were performed. All experiments were assigned into groups including relevant controls and analysis was done objectively and without bias.

Reporting for specific materials, systems and methods

We require information from authors about some types of materials, experimental systems and methods used in many studies. Here, indicate whether each material, system or method listed is relevant to your study. If you are not sure if a list item applies to your research, read the appropriate section before selecting a response.

Materials & experimental systems

n/a	Involved in the study	
☐	☒	Antibodies
☐	☒	Eukaryotic cell lines
☒	☐	Palaeontology
☐	☒	Animals and other organisms
☒	☐	Human research participants
☒	☐	Clinical data

Methods

n/a	Involved in the study	
☒	☐	ChIP-seq
☐	☒	Flow cytometry
☒	☐	MRI-based neuroimaging

Antibodies

Antibodies used

G3BP1 (Homemade)
Tubulin (Developmental Studies Hybridoma Bank)
Lamin B1 (Abcam Cat# ab16048)
RB (Abcam Cat# ab181616)
pRB (S807/811) (Cell Signaling Technology Cat# 8516)
p53 (Millipore Cat# OP43-100UG)
pp53 (S15) (Cell Signaling Technology Cat# 9284)
p21 (Abcam Cat# ab7960)
p16 (Abcam Cat# ab108349)
p65 (Abcam Cat# ab16502)
STAT3 (Cell Signaling Technology Cat# 9139)
pSTAT3 (Y705) (Cell Signaling Technology Cat# 9145)
IκBα (Cell Signaling Technology Cat# 4814)
pIκBα (S32) (Abcam Cat# ab92700)
IRF3 (Cell Signaling Technology Cat# 11904)
pIRF3 (S396) (Cell Signaling Technology Cat# 4947)
H3K9me3 (Abcam Cat# b8898)
Phospho-Histone H2A.X (Ser139/Tyr142) (Cell Signaling Technology Cat# 5438)
cGAS (Thermo Fisher Scientific Cat# 14-5158-82)
Ki-67 (Abcam Cat# ab16667)

Validation

The antibody against G3BP1 was validated through siRNA knockdown to confirm the specificity.

The antibody against Tubulin (Developmental Studies Hybridoma Bank) was not validated

Antibodies purchased from Abcam were validated as per their website stating "Antibodies are validated in western blot using lysates from cells or tissues that we have identified to express the protein of interest. Once we have determined the right lysates to use, western blots are run and the band size is checked for the expected molecular weight. We will always run several controls in the same western blot experiment, including positive lysate and negative lysate. When possible, we also include knock-out (KO) cell lines as a true negative control for our western blots. We are always increasing the number of KO-validated antibodies we provide. In addition, we run old stock alongside our new stock. If we know the old stock works well, this also acts as a suitable positive control. If the western blot result gives a clear clean band and we are happy with the result from the control lanes, these antibodies will be passed and added to the catalog. "

Antibodies purchased from Cell Signaling Technology were validated as per their website stating "Antibody signal is measured in model systems with known presence/absence of target signal. Includes wild-type vs. genetic knockout, targeted induction or silencing."

Antibodies purchased from Thermofisher were validated using genetic knockdown/ablation or RNAi as per their website.

Antibodies purchased from Millipore were validated using genetic knockdown/ablation or RNAi as per their website.

Eukaryotic cell lines

Policy information about cell lines

Cell line source(s)

WI-38 cells were purchased from the European Collection of Authenticated Cell Cultures (ECACC Cat# 90020107)
IMR-90 cells were purchased from the American Type Culture Collection (ATCC Cat# CCL-186)
HEK 293T cells were purchased from the American Type Culture Collection (ATCC Cat# CRL-3216)
A549 (ATCC Cat# CCL-185) and Immortalized Skin Fibroblasts (CBSF-4T) were kindly gifted to us from the laboratory of Dr. Christian Beausejour at the CHU Sainte-Justine Research Center

Authentication

All ATCC cell lines (WI-38, HEK293T and A549 cells) were authenticated by ATCC using morphology, karyotyping, and PCR based approaches to confirm the identity of human cell lines and to rule out both intra- and interspecies contamination. These include an assay to detect species specific variants of the cytochrome C oxidase I gene (COI analysis) to rule out inter-species contamination and short tandem repeat (STR) profiling to distinguish between individual human cell lines and rule out intra-species contamination

All ECACC cell lines (IMR-90) were authenticated using short tandem repeat (STR) profiling to distinguish between individual human cell lines and rule out intra-species contamination

CBSF-4T cells were not authenticated

Mycoplasma contamination

Previously tested by distributors and routinely tested using DAPI. All cell lines used were routinely tested as described and were negative for Mycoplasma.

Commonly misidentified lines
(See ICLAC register)

No cell lines used are listed in the database of commonly misidentified cell lines.

Animals and other organisms

Policy information about studies involving animals; ARRIVE guidelines recommended for reporting animal research

Laboratory animals	NOD/SCID/IL2Rγ null (NSG) Mice<br>- 20-24 degrees celsius<br>- 40-70% humidity (typically 50%)<br>- 12 hour dark/light cycle
Wild animals	This study did not involved wild animals
Field-collected samples	No field collected samples were used in the study.
Ethics oversight	All in vivo manipulations were previously approved by the CHU Sainte-Justine Research Centre institutional committee for good laboratory practices for animal research (Protocol #579).

Note that full information on the approval of the study protocol must also be provided in the manuscript.

Flow Cytometry

Plots

Confirm that:

☒ The axis labels state the marker and fluorochrome used (e.g. CD4-FITC).

☒ The axis scales are clearly visible. Include numbers along axes only for bottom left plot of group (a 'group' is an analysis of identical markers).

☒ All plots are contour plots with outliers or pseudocolor plots.

☒ A numerical value for number of cells or percentage (with statistics) is provided.

Methodology

Sample preparation	Senescent WI-38 cells were seeded at 70–80% confluence in 6 cm culture plates, cultured and induced into senescence using 10Gy of irradiation or through transduction of HRAS using lentivirus. Eight days post-irradiation or transduction, lysosomal alkalinisation was induced by pre-treating cells with 100nM bafilomycin A1 for 1 h in fresh cell culture medium at 37°C and 5% CO2. C12FDG (33µM) solution was then added to the cell culture medium for 2 h. The cells were harvested by trypsinization and resuspended 1X PBS. The cells were analyzed by flow cytometry within 1 h.
Instrument	BD FACSCanto™ II
Software	FACSDiva 8 was used during collection<br>FlowJo Version10.5.2 was used to analyze Flow Cytometry Data
Cell population abundance	No cell sorting was used to determine abundance of cell population since cultured WI-38 cells were used for flow cytometry from a pure population.
Gating strategy	To estimate relative SA-β-Gal activity, a two-parameter display of FSC vs. SSC was set up excluding subcellular debris. Single cells were selected using a two-parameter display of FSC-H vs. FSC-A. Non-labeled samples were used to determine auto-fluorescence.

☒ Tick this box to confirm that a figure exemplifying the gating strategy is provided in the Supplementary Information.
